# Approaches for automized expansion and differentiation of human MSC in specialized bioreactors

**DOI:** 10.1186/1753-6561-7-S6-P47

**Published:** 2013-12-04

**Authors:** Anne Neumann, Antonina Lavrentieva, Dominik Egger, Tim Hatlapatka, Cornelia Kasper

**Affiliations:** 1 Department for Biotechnology, University of Natural Resources and Life Sciences, 1190 Vienna, Austria; 2 Institute for Technical Chemistry, Leibniz University of Hannover, 30167 Hanover, Germany

## Background and experimental approach

A main challenge in cell therapies and other tissue regeneration approaches is to produce a therapeutically significant cell number. For expansion of mesenchymal stem cells (MSC) the cultivation on 2D plastic surfaces is still the conventional procedure, even though the culture conditions differ significantly from the 3D environment in vivo. Additionally, static amplification of MSC is a labour-intensive procedure. We therefore used a specialized rotating bed bioreactor in order to maximize ex vivo expansion of MSC. MSC were isolated from umbilical cord (UC) by explant method approach under xeno-free conditions. UC-MSC were thereafter expanded under dynamic conditions in a novel rotating bed bioreactor. The bioreactor system was designed to enable integration of sensors for online monitoring of various parameters (e.g. pH, pO_2_, pCO_2_) and hence, allow ensured cultivation under well controlled and reproducible conditions. Beside cell expansion, directed differentiation of MSC was also achieved in bioreactors. MSC lack the ability to grow in 3D direction and build functional tissue in vitro. Thus, it is necessary to seed and culture MSC on 3D matrices to obtain functional implants. For guided differentiation towards the osteogenic lineage, MSC were cultivated on ceramic porous matrices under dynamic conditions. Custom-made miniaturized perfusion bioreactors for parallel testing were designed and optimized for that purpose.

## Methods

MSC isolation was achieved as described previously [1]. Briefly, umbilical cord tissue is cut into pieces (approx. 0.5 cm^2^) and cultivated for 10 days in αMEM containing 15% human serum in cell culture flasks. Cells grow out of the tissue pieces and adhere to the cell culture plastic. Subsequently, cells are harvested and subcultivated in αMEM containing 10% human serum.

**Figure 1:**
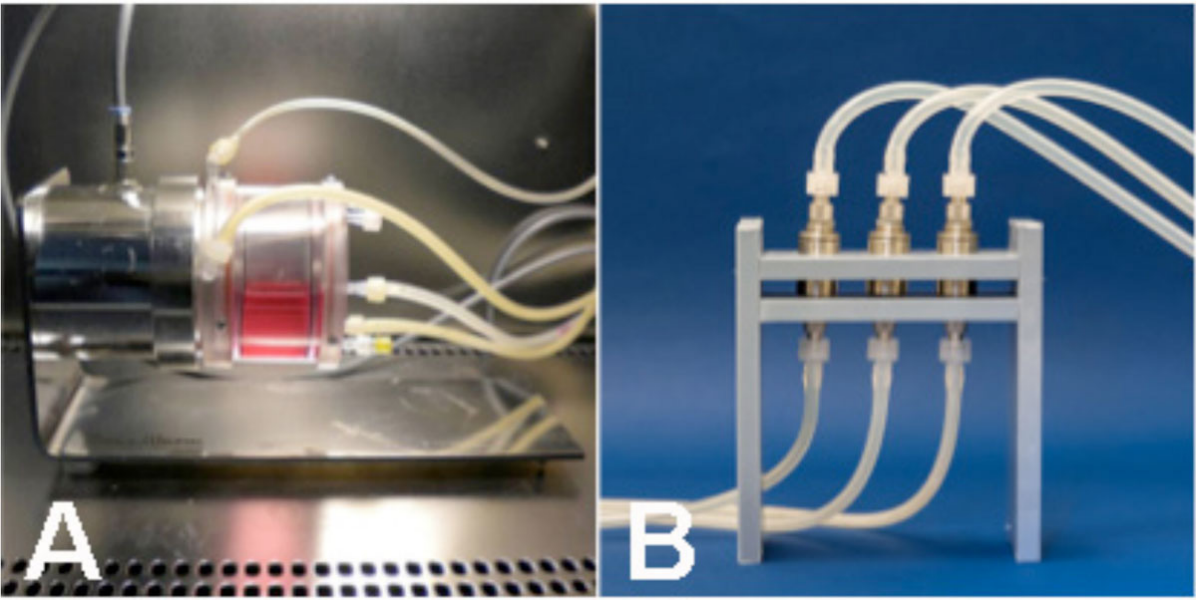
**A) Rotation bed bioreactor for expansion of MSC and B) Perfusion bioreactors**.

UC-MSC were expanded in a rotating bed bioreactor (Figure 1A). The bioreactor chamber is a cylindrical bioreactor shell, comprising an inlet (bed) fixed to a magnet whereas the bioreactor chamber is hold by that magnet to an engine. The inlay is rotating, while the shell is fixed. The inlay comprises cell culture plastic slides with an all over surface of 2000 cm^2^, requiring approximately 130 ml cell culture medium to be completely covered. The bioreactor is equipped with a feed circulation for fresh medium and removal of waste. An additional circulation to pH and pO_2 _sensor electrodes enables online monitoring. Sampling is performed through a septum in the bioreactor shell. Gas mixture of air and CO_2 _is supplied by an overlay stream. The whole bioreactor set up is situated in a GMP conform breeder, enabling sterile handling as well as an environmental temperature of 38°C. The system is connected to a control unit, which comprises gas regulation, pumps and software for parameter set up and monitoring. UC-MSC were seeded (1,500 cells/cm^2^) in the bioreactor for 24 h hours and expanded for 5 days under dynamic conditions. Medium feed was adjusted depending on glucose consumption. After 5 days of cultivations UC-MSC were harvested by flushing the bioreactor with accutase for 20 min. MSC were counted, examined regarding their senescence (β-galactosidase), proliferation capacity (glucose/lactate) and differentiation potential (Oil Red O, Alizarin Red, Von Kossa, Alcian Blue), as well as surface markers.

Perfusion bioreactors consist of a stainless steel tube in which the material is inserted and a piston, which closes the reactors (Figure 1B). As the piston can be adjusted in height a bioreactor can host materials with a diameter of 10 mm and a high of max. 10 mm. MSC-seeded ceramic materials (10 mm × 3 mm) were inserted into the bioreactor. The bioreactors are connected to a medium reservoir, equipped with a sterile filter for gas exchange. The volume of the bioreactor containing the ceramic material is 1,5 ml, the overall volume of medium used for the cultivation is 10 ml. Dynamic cultivation was achieved using flow rates of 0.3 and 1.5 ml/min. Viability was examined using MTT Assay. Cell distribution throughout the scaffold was investigated using DAPI staining. The status of differentiation was examined using different histological stainings (e.g. Von Kossa, Calcein, Alizarin Red).

## Results and discussion

UC-MSC isolated using explant method approach fulfils MSC criteria, such as adherence to plastic surfaces, specific surface marker pattern and differentiation potential towards at least the adipogenic, chondrogenic and osteogenic lineage [1].

MSC expanded under dynamic conditions in a rotating bed bioreactor also fulfil these MSC criteria. Furthermore it could be shown, that MSC consume glucose and produce lactate during dynamic cultivation in the rotating bed bioreactor and consequently proliferate. After 5 days of cultivation MSC were investigated regarding their specific surface marker. They express CD44, CD73, CD90 and CD 105 and lack CD 31, CD34 and CD45.

MSC on ceramic materials could be shown to differentiate towards the osteogenic lineage under static conditions. Also after dynamic cultivation with a medium perfusion of 0.3 ml/min and even 1.5 ml/min cells adhere on the macro porous ceramic material, were viable and equally distributed throughout the scaffold. Seeding efficiency was found to be approximately 20%. Osteogenic differentiation could be achieved by cultivation in perfusion bioreactors.

## Conclusion

MSC could be successfully isolated from human umbilical cord tissue. MSC expansion in the rotation bed bioreactor provides a high number of cells, maintaining their stem cell properties such as specific surface markers, proliferation capacity and differentiation potential. Cultivation of MSC in perfusion bioreactors have been shown to support and improve osteogenic differentiation as mechanical plays an important role in directing MSC fate. Our results support the argument that the application of tailor-made bioreactors are an essential step toward the production of stem cell based therapeutics and tissue engineering products.
